# Hemodynamic Segmentation of Brain Perfusion Images with Delay and Dispersion Effects Using an Expectation-Maximization Algorithm

**DOI:** 10.1371/journal.pone.0068986

**Published:** 2013-07-19

**Authors:** Chia-Feng Lu, Wan-Yuo Guo, Feng-Chi Chang, Shang-Ran Huang, Yen-Chun Chou, Yu-Te Wu

**Affiliations:** 1 Department of Biomedical Imaging and Radiological Sciences, National Yang-Ming University, Taipei, Taiwan, ROC; 2 Brain Research Center, National Yang-Ming University, Taipei, Taiwan, ROC; 3 Department of Physical Therapy and Assistive Technology, National Yang-Ming University, Taipei, Taiwan, ROC; 4 Department of Education and Research, Taipei City Hospital, Taipei, Taiwan, ROC; 5 Department of Radiology, Taipei Veterans General Hospital, Taipei, Taiwan, ROC; 6 Faculty of Medicine, National Yang-Ming University, Taipei, Taiwan, ROC; University of Adelaide, Australia

## Abstract

Automatic identification of various perfusion compartments from dynamic susceptibility contrast magnetic resonance brain images can assist in clinical diagnosis and treatment of cerebrovascular diseases. The principle of segmentation methods was based on the clustering of bolus transit-time profiles to discern areas of different tissues. However, the cerebrovascular diseases may result in a delayed and dispersed local perfusion and therefore alter the hemodynamic signal profiles. Assessing the accuracy of the segmentation technique under delayed/dispersed circumstance is critical to accurately evaluate the severity of the vascular disease. In this study, we improved the segmentation method of expectation-maximization algorithm by using the results of hierarchical clustering on whitened perfusion data as initial parameters for a mixture of multivariate Gaussians model. In addition, Monte Carlo simulations were conducted to evaluate the performance of proposed method under different levels of delay, dispersion, and noise of signal profiles in tissue segmentation. The proposed method was used to classify brain tissue types using perfusion data from five normal participants, a patient with unilateral stenosis of the internal carotid artery, and a patient with moyamoya disease. Our results showed that the normal, delayed or dispersed hemodynamics can be well differentiated for patients, and therefore the local arterial input function for impaired tissues can be recognized to minimize the error when estimating the cerebral blood flow. Furthermore, the tissue in the risk of infarct and the tissue with or without the complementary blood supply from the communicating arteries can be identified.

## Introduction

The advent of modern imaging modalities has enabled studies to unravel temporal hemodynamic patterns in different regions of the brain to assist in the assessment of cerebrovascular diseases. Dynamic-susceptibility-contrast MR (DSC-MR) imaging records signal changes associated with different blood supply patterns following the intravenous injection of a bolus of contrast agent. Based on the bolus profile in the arterial compartment and on indicator dilution theory [1], [2], computations of cerebral hemodynamic parameters, such as relative cerebral blood volume (rCBV), relative cerebral blood flow (rCBF), mean transit time (MTT), and time to peak (TTP), are possible. Hemodynamic parameter maps have been intensively used in clinical applications, including the assessment of brain tumors [3]–[5], brain ischemia [4]–[7], occlusive cerebrovascular disease [8], and radiation necrosis [3], [5].

Classification of brain hemodynamics into different components is essential to facilitate the analysis and assessment of brain perfusion. In our previous studies, fast independent component analysis (FastICA) [9], [10], the noiseless independent factor analysis (NIFA) [11], and analysis based on expectation-maximization (EM) of a mixture of multivariate Gaussians (MoMG) [12] were developed to discern brain tissues. In the EM-MoMG method, the data was similarly zero-mean normalized and reduced by principal component analysis (PCA) [13]. Under the assumptions that the distribution of the reduced data associated with each tissue type was multivariate Gaussian distributed and that the overall distribution of the reduced data was a MoMG [14], alternating iterative expectation (E) and maximization (M) steps [15] were performed. The E-steps calculated the expected log-likelihood conditions using the observed data along with the currently estimated model parameters. The M-steps updated the model parameters by maximizing the log-likelihood values. During this EM process, the posterior probabilities in relation to each tissue type were obtained at each pixel, which in turn provided sufficient statistical validity, i.e., maximal probability, to determine the tissue type of each pixel. As a consequence, all tissue compartments could be segmented out simultaneously using the EM-MoMG method, instead of the one-by-one segmentation process used in the FastICA method. However, the EM-MoMG method required a good initial guess of the model parameters to achieve satisfactory results. In this study, we improved the EM method by using the results of hierarchical clustering (HC) on whitened perfusion data as initial parameters for a MoMG.

The segmentation of brain tissues using perfusion images relies on the clustering of bolus transit-time profiles to discern areas of different tissue types. However, cerebrovascular diseases would change the local perfusion of the impaired tissues with different levels of delay and dispersion, and therefore complicate the overall distribution of the signal profiles. The delay and dispersion phenomena were resulted from the defect of the blood vessel structures, such as the internal carotid artery (ICA) stenosis, moyamoya disease, and arteriovenous malformation. Assessing the accuracy of the segmentation technique under delay/dispersed circumstance is critical to accurately evaluate the severity of the vascular disease and locate the impaired tissues. However, previous literatures have focused on the effects of delay and dispersion in the deconvolution algorithm for estimating the rCBF [16]–[18], but not the effects in the tissue segmentation and impaired tissue recognition. In this study, Monte Carlo simulations were conducted to evaluate the performance of proposed method under different levels of delay, dispersion, and noise of signal profiles in tissue segmentation. Finally, we analyzed the perfusion data from five normal participants, a patient with unilateral ICA stenosis, and a patient with moyamoya disease using the proposed method.

## Materials and Methods

### Tissue Segmentation using the HC-EM-MoMG Method

Brain regions were extracted from perfusion images using Otsu’s method [19], followed by an erosion and dilation operation [20]. The brain region for each image was assumed to comprise 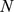 pixels, and the observation of 65 temporal images was represented by a 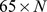 matrix. The dimension of each data set was reduced by PCA [13] from 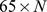 to 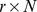. During PCA, at least 99% of the data variance was retained.

Similar to PCA, the data whitening process first calculates eigenvalues and corresponding eigenvectors for the covariance matrix of the zero-mean normalized data. The zero-mean data were then transformed via a whitening matrix, which was constructed of eigenvectors and eigenvalues, so that the covariance matrix became an identity matrix [13]. The compressed, transformed data resulting from whitening dimension reductions were subsequently used in HC.

During HC, the compressed data are denoted by a matrix 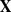 with size 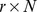 and each 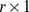 column vector in 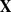 is referred to as a feature vector, *x*. Initially, HC was carried out in the data set 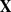 based on a created cluster tree with a multilevel hierarchy in which any two nearby clusters at one level become merged into one cluster at the next higher level. The HC algorithm comprised three major parts. First, each group contains a single feature vector of 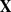, i.e., 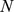 groups in 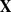, and the element in the dissimilarity matrix is the squared Euclidean distance between any two featured column vectors in 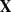, which was defined by
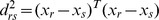
where 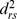 is the Euclidean distance between two featured column vectors 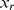 and 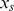. Second, in order to construct the cluster tree, we employed the Ward’s method [21] to measure the distance between two clusters which was defined by



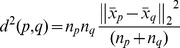
where 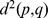 is the distance between two clusters of featured vectors, 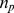 and 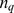 are the numbers of featured vectors in cluster *p* and *q*, respectively, 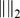 is the Euclidean distance, 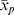 and 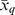 are the centroids of clusters *p* and *q*, respectively, which are defined by



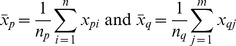
where 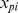 is the *i*th featured vector in cluster *p* and 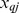 is the *j*th objects in cluster *q*. The two clusters with the smallest between-group distance are grouped together to form a new group. The algorithm proceeds until all of the feature vectors fall within a single group, thus forming a hierarchical clustering tree. Third, the level or scale of clustering is determined by cutting the hierarchical cluster tree. The HC results for the condensed data, 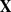, were used to be the initial guess of the mean vectors, the covariance matrices of the MoMG, and the proportion of each tissue class for the subsequent EM estimation [12].

The E-step of EM estimation is to compute the posterior probabilities of tissue classes *p(i|x_n_,θ^j−1^)* based on the estimation from previous *j-*1^th^ iteration:
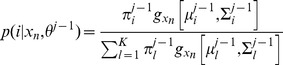
Where i = 1,…,K represent labels of tissue classes, 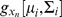 represents the multivariate Gaussian density with the mean vector 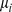 and covariance matrix 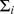 of tissue class *i*, 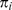 denotes the proportion of each class i, and 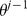 denotes the parameters 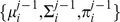 at the *j*-1^th^ iteration. The M-step consists of estimating parameters 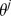 as follows (please see Appendix in [12] for details):



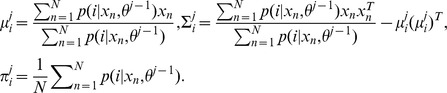



Once all tissues of interest were identified, averaged signal-time curves for each tissue type were computed and the arterial input function (AIF) was modeled from the averaged concentration-time curve of artery in order to compute the rCBV, rCBF, and MTT of each segmented tissue types [22]–[24]. Specifically, we first computed the concentration-time curve 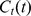 for each pixel using the formula:
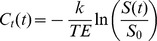
where 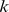 is a constant, 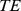 is the echo time, and 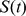 and 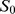 are the signal intensities of each pixel at time 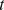 and at the baseline, respectively. By using the indicator dilution theory, one can determine the rCBV for each pixel as a ratio of the area integrating over the first pass of the contrast agent under the concentration-time curve, 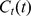, to that under the AIF, 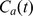,



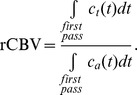



The rCBF can be computed based on the relationship with concentration-time curve for each pixel:



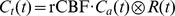
where 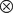 denotes convolution, · denotes multiplication, and 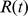 is the residue function for the pixel. The 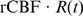 curve for each pixel can be resolved using the singular value decomposition (SVD) method and the value of rCBF at each pixel was determined by the maximum value of 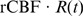 curve [25]. Finally, the MTT of contrast-agent particles passing through a pixel was defined as



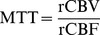



### Design of Hypothetical Compartments for Monte Carlo Simulations

**Figure 1:**
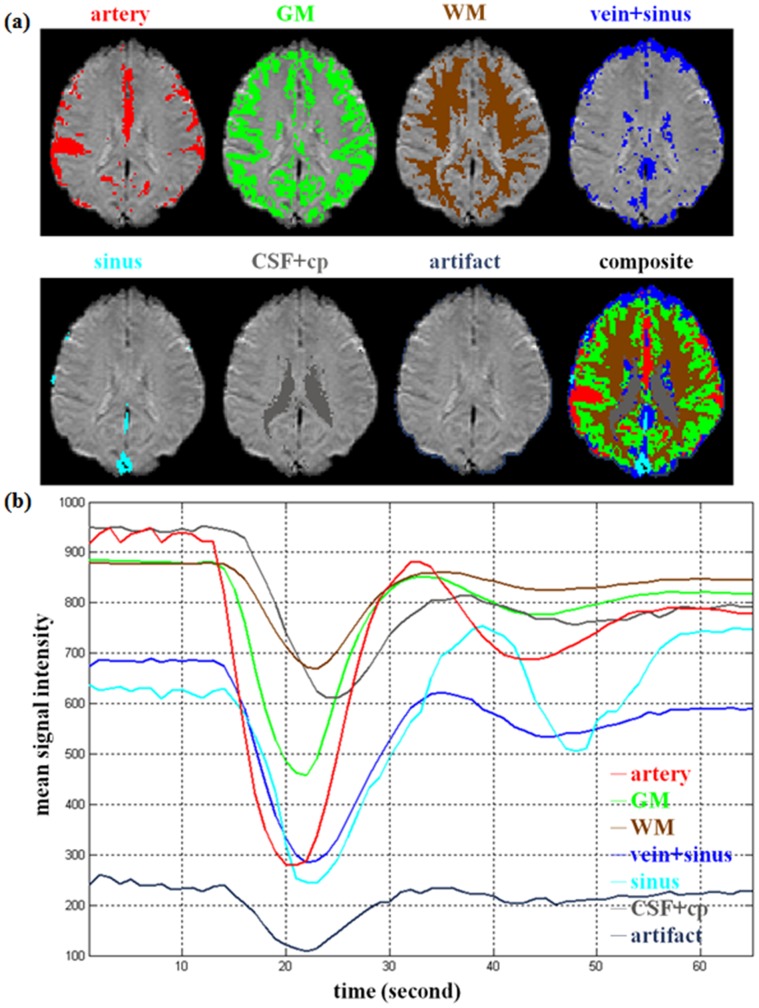
An example of seven hypothetical tissue clusters and the mean signal-time curves. The number of pixels and intensity distribution of each hypothetical cluster were generated based on the ROI on one of the raw data sets.The selected ROIs on the raw data represented artery (551 pixels), GM (1741 pixels), WM (1636 pixels), vein and sinus (610 pixels), sinus (80 pixels), CSF and cp (412 pixels), and artifact (175 pixels), respectively. The averaged signal-time curves (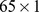), and covariance matrices of intensities across time (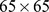) within ROIs on the raw data were employed as the hypothetical parameters for the MoMG model to create a set of noise-free simulated dynamic images using random number generators.

Simulated dynamic images based on a region of interest (ROI) in one of the raw data sets were adopted. In those images each simulated data set accommodated the possibilities of having four to nine hypothetical clusters (Fig. 1). Pixels within each hypothetical tissue area were assumed to be spatially independent, and pixel intensities across time were assumed to be multivariate Gaussian-distributed. The averaged signal-time curves, each of which was a 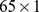 vector, and the covariance matrices of intensities across time, each of which had a dimension of 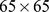, within ROIs in the raw data were employed as hypothetical parameters for the MoMG model to create sets of noise-free simulated dynamic images using random number generators. In addition, Gaussian noise was added to each set of noise-free simulated dynamic images to produce the signal to noise ratio (SNR) levels of 40 and 70. For each of the hypothetical clusters and each noise level, a Monte Carlo simulation comprising 1000 runs was performed. Accordingly, the total number of simulated image sets was 6 (number of hypothetical clusters) 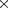
3 (noise levels) 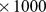
(repetitions for each combination) = 18000. Each set of simulated dynamic images was rearranged into a 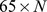 matrix before classification, where 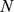 (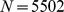 in the simulation) was the number of pixels representing the brain area.

### Monte Carlo Simulations for EM-MoMG with Differenet Initializations

The parameters of MoMG for EM segmentation were initialized by 2 different means: 1) HC results from whitened data; 2) random sampling of columns from the matrix representing the PCA data, which was used in our previous EM-MoMG method [12]. The number of tissue classes to be tested ranged from 4 to 9. After comparing posterior probability values at each pixel, the cluster resulting in the maximum probability was identified, that pixel was then labeled accordingly. The classification rate for each cluster, denoted by 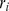, was defined by the observed proportion of agreements, i.e., 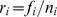, where 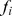 was the number of agreements for the *i* ^th^ cluster and 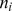 was the total number of pixels in the *i* ^th^ class. The classification rate for a set of simulated dynamic images, denoted by 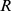, was computed by 
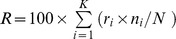
 (as a percentage), where 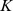 is the number of clusters. No delay and dispersion effects were added to these simulated perfusion images.

### Monte Carlo Simulations with Delay and Dispersion

**Figure 2:**
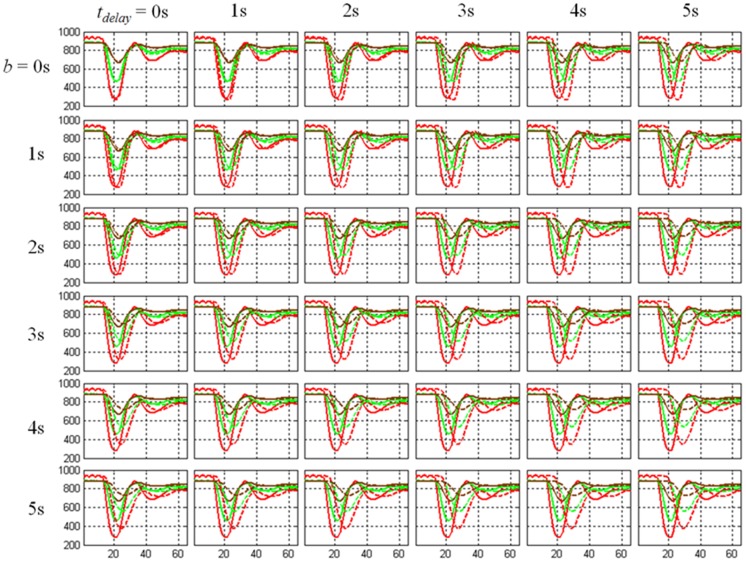
The effects of delay and dispersion. Each row represents the delay effects (*t_delay_*_ = _0, 1, 2, 3, 4, 5 s) with a mixture of the dispersion effects in each column (*b = *0, 1, 2, 3, 4, 5 s) for the signal-time curves of artery (red), GM (green), and WM (brown). The solid curves stand for the normal artery, GM and WM, and the dashed curves depict the corresponding delayed and dispersed versions.

**Figure 3:**
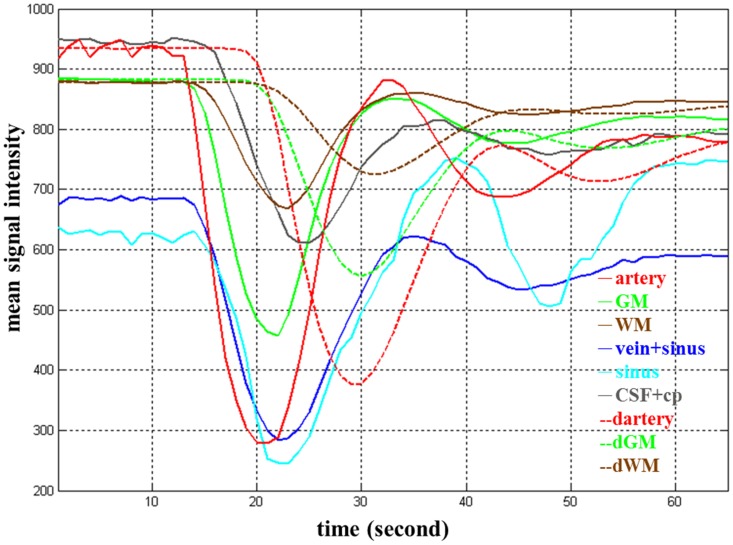
Simulated mean signal-time curves for nine tissue types. The curves of dArtery, dGM and dWM were simulated with *t_delay = _*5 s *and b = *5 s compared to their normal counterparts, namely, artery, GM and WM.

Delay and dispersion are two popular phenomena in the hemodynamics of subjects with cerebrovascular diseases. Thus, Monte Carlo simulations were conducted to investigate the influences of these two factors on the proposed HC-EM-MoMG method. The concentration-time curves, 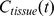, for anomalous tissue types were created using the following formula [22]

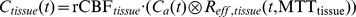
where the effective residue function 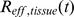 [18] is given by



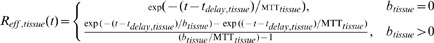
in which 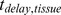 and *b_tissue_* denote time delays relative to AIF and dispersion, respectively. The values of 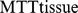, 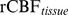, 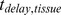, and *b_tissue_* were estimated to simulate the concentration-time curves of the delayed/dispersed artery (dArtery), delayed/dispersed gray matter (dGM), and delayed/dispersed white matter (dWM). Specifically, we calculated the values of rCBF and MTT for artery, GM, and WM, based on the raw data of hypothetical compartments. The normal rCBF and MTT values of artery, GM, and WM were also used for dArtery, dGM and dWM, respectively. The derived simulated concentration-time curves were then converted into signal-time curves using the following equation
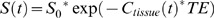
where *S_0_* is the baseline signal and TE is echo time. In addition to the signal-time curves for dArtery, dGM, and dWM, signal-time curves for artery, GM, WM, vein combined with sinus (vein+sinus), sinus, as well as for CSF including the corpus plexus (CSF+cp) tissues were also used in these simulations. Hence, there were 9 hypothetical tissue types in the test of delay and dispersion effects. With *t_delay_* values of 0, 1, 2, 3, 4, and 5 s and dispersion *b* values of 0, 1, 2, 3, 4, and 5 s, overall 36 combinations of abnormal conditions were simulated (Fig. 2). The number of voxels in the impaired components, i.e., dArtery, dGM and dWM, were designated to be 50%, 25%, or 13% of the total voxels in the artery, GM and WM components. Noise-free and SNR levels of 40 and 70 were created. The total number of simulated data sets was 6 (different *t_delay_*’s) 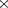
6 (different *b*’s) 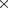
3 (different numbers of voxels in abnormal components) 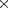
3 (noise levels) 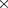
1000 (repetitions for each combination) = 324000. Fig. 3 illustrates the 9 simulated mean signal-time curves in which the time settings of *t_delay_* and *b* for the impaired compartments were both 5 s.

### Participants and Data Recording

This study received prior approval from the Institutional Review Board of Taipei Veterans General Hospital. Each participant provided written informed consent before participating in this study. Five healthy volunteers (3 males and 2 females) aged from 18 to 47 years, one 78 year-old male with a unilateral ICA stenosis, and one 57 year-old female with moyamoya disease participated in this study. A multi-slice gradient-echo echo-planar-imaging pulse sequence on a 1.5 Tesla scanner (Signa CV/*i*, GE Medical Systems, Milwaukee, WI, USA) was used to acquire dynamic perfusion images. For the healthy subjects, trans-axial imaging was used with TE/TR = 60/1000 ms, flip angle = 90 degrees, FOV = 24 cm ×24 cm, matrix = 128×128, slice thickness/gap = 5/5 mm for 7 slices, one acquisition, and 70 images per slice location with a one second temporal resolution. Twenty ml of Gd-DTPA-BMA (Omniscan®, 0.5 mmol/ml, Nycomed Imaging, Oslo, Norway) followed by 20 ml of normal saline were delivered administratively using a power injector (Spectris®, Medrad, Indianola, PA, USA) at a flow rate of 3–4 ml/s in the antecubital vein. Some imaging protocol settings were changed for the ICA patient, i.e., TE/TR = 40/1000 ms, flip angle = 60 degrees, slice thickness/gap = 7/14 mm for 7 slices. All routines were implemented using MATLAB (MathWorks, Inc., Natick, MA, USA) codes and carried out on a 2.66 GHz Intel-Core2-based personal computer.

## Results

### Simulation Results for Different Initializations of EM-MoMG

Table 1 presents the average classification rates
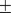
standard deviations (in percentages) for Monte Carlo simulations obtained from the EM-MoMG method initialized by 2 different approaches. The EM-MoMG method initialized by the results from HC on whitened data outperformed the initialization approach using the random sampling on PCA data. The average classification rates were higher using HC on whitened data (between 99.0
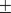
0.39% for noise-free conditions with four hypothetical tissue clusters and 93.6
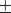
1.42% for the condition of SNR = 40 with 9 hypothetical tissue clusters, Table 1). The standard deviations of classification rates were also smaller (always less than 1.42%) using the HC results on whitened data as initialization, thereby implying the HC-EM-MoMG method can produce accurate and reliable segmentations.

**Table 1:** The averaged classification rates
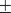
standard deviations (in percentage) for Monte Carlo simulations resulted from the EM-MoMG method with 2 different initials.

		Number of hypothetical tissue clusters					
SNR	Initials methods	4	5	6	7	8	9
NF*	HC results of whitened data	99.0 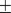 0.39	97.0 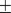 0.72	96.7 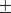 0.86	96.3 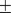 0.93	95.1 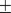 1.31	94.1 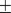 1.35
	Random sampling on PCA data	95.7 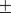 12.48	96.4 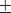 4.28	92.9 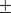 17.48	91.0 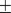 18.86	87.9 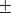 18.01	89.1 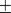 19.13
70	HC results of whitened data	99.0 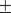 0.40	96.9 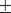 0.74	96.5 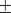 0.88	96.1 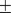 0.95	95.0 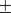 1.30	93.9 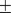 1.38
	Random sampling on PCA data	95.3 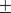 14.11	95.5 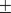 9.77	92.5 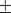 18.00	91.0 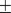 18.60	87.0 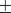 19.90	88.8 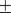 19.28
40	HC results of whitened data	98.9 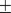 0.40	96.7 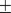 0.75	96.2 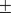 0.93	95.9 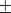 0.99	94.8 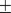 1.34	93.6 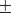 1.42
	Random sampling on PCA data	95.2 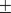 14.05	95.7 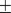 5.26	93.1 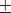 16.49	90.5 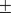 19.88	86.7 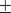 19.63	88.8 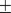 18.87

* NF: noise-free.

The mean classification rates from all initialization methods decreased slightly with the change from the noise-free condition to SNR = 40. In the case of the proposed HC-EM-MoMG method, the minimal mean classification rate and maximal standard deviation were 93.6% and 1.42%, respectively, in the 9 hypothetical tissue clusters with SNR = 40 (Table 1). The classification performances degraded as the number of hypothetical tissue clusters increased. In the worst case, i.e., SNR = 40, the results obtained from the HC-EM-MoMG method were from 98.9
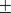
0.40% to 93.6
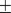
1.42%.

In addition, we computed minimal description length (MDL) [26] values when different numbers of tissue classes were used in the EM-MoMG method with HC on whitened data under noise-free, and SNR = 70, and 40 conditions. The results indicated that the MDL values were minimal when the number of tissue classes was identical to the hypothetical ones.

### Simulation Results with Various Delays, Dispersions, and Percentages of Abnormal Voxels

Table 2 shows the simulation results with different delays and dispersions under various noise conditions. The results showed that the classification rates increased as either delay or dispersion increased. For example, the classification rates under noise-free conditions increased from 71.0
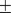
5.32% to 89.9
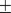
5.92% when the delay increased from 1 s to 5 s, and increased from 76.4
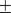
5.87% to 87.3
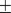
4.36% as dispersion increased from 1 s to 5 s. In general, the classification rates were satisfactory when the differences in either delay or dispersion between normal and corresponding impaired compartments were longer than one second. As examples, the classification rates were mostly over 80% when (*b*, *t_delay_*) = (0, 2), (1, 1), (2, 0), and were over 85% when (*b*, *t_delay_*) = (0, 3), (1, 2), (2, 1), (3, 0). The results also show that as noise increased from the noise-free condition to SNR = 40, the mean classification rates also decreased. As examples, the mean classification rates decreased from 87.0
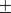
7.94% with noise-free condition to 86.6
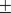
6.55% with SNR = 40 at (*b*, *t_delay_*) = (5, 5). In addition, the mean classification rates obtained from the six normal tissues and the three impaired compartments (dArtery, dGM, dWM) under different levels of delay and dispersion were 4% to 20% lower than the rates from the 9 normal tissues shown in Table 1. This result suggested that some of the perfusion profiles of the impaired compartments may be equivocal and more difficult to distinguish. However, as delay and dispersion were both longer than one second, the mean classification rate was greater than 85%.

**Table 2:** The averaged classification rates
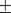
standard deviations (in percentage) for Monte Carlo simulations resulted from the EM-MoMG method initialized by the results of HC on the whitened simulated data with different delayed time, dispersion and various SNRs.

		Delay					
SNR	Dispersion	0 s	1 s	2 s	3 s	4 s	5 s
**NF** *	**0** **s**	–	71.0 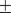 5.32	80.5 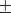 5.70	85.5 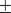 4.86	88.0 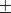 4.83	89.9 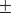 5.92
	**1** **s**	76.4 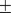 5.87	83.7 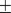 5.40	86.4 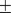 5.51	89.0 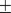 4.89	89.3 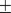 5.46	88.4 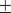 6.96
	**2** **s**	83.0 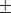 5.18	85.9 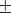 4.71	88.1 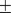 4.88	88.1 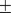 5.66	87.7 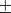 7.13	86.7 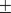 8.71
	**3** **s**	85.3 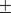 5.06	87.1 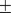 4.65	89.0 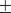 5.00	87.6 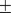 7.07	87.6 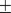 6.85	87.8 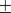 7.57
	**4** **s**	86.8 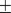 4.87	87.7 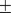 4.40	88.6 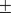 5.11	87.8 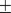 6.15	87.3 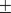 7.60	87.3 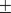 7.62
	**5** **s**	87.3 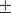 4.36	88.8 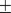 4.88	89.0 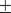 5.92	87.6 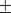 7.13	87.1 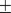 7.43	87.0 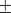 7.94
**70**	**0** **s**	–	69.2 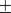 4.84	77.7 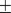 5.63	85.0 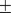 5.14	87.3 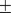 5.62	88.2 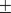 6.25
	**1** **s**	75.0 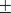 5.14	82.8 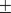 4.24	86.0 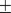 5.16	88.6 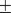 5.11	88.8 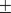 5.92	86.3 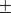 7.33
	**2** **s**	82.1 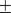 4.82	85.3 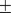 5.05	87.4 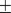 4.57	88.9 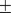 5.49	87.1 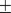 6.01	86.7 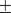 6.99
	**3** **s**	85.2 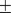 4.52	86.3 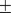 4.65	87.4 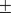 4.59	87.7 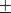 6.50	86.8 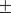 7.38	87.3 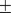 6.83
	**4** **s**	85.6  4.94	86.4 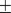 4.39	87.8 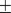 5.38	86.5 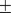 6.26	86.6 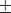 7.47	86.5 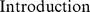 7.53
	**5** **s**	86.6 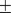 4.62	87.3 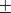 4.68	87.6 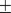 5.77	87.8 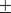 6.50	86.6 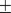 7.18	86.7 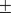 6.90
**40**	**0** **s**	–	67.5 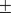 4.65	75.8 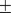 5.51	82.6 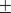 4.71	86.2 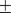 5.35	87.8 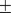 5.76
	**1** **s**	72.4 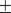 5.08	80.1 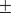 5.09	84.3 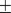 5.04	87.3 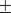 5.02	86.8 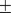 6.11	85.4 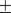 6.80
	**2** **s**	79.2 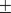 4.14	83.3 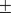 4.93	85.3 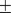 4.89	86.5 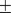 5.29	86.5 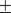 6.51	85.6 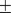 7.29
	**3** **s**	82.6 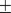 4.77	84.6 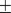 4.28	86.7 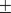 5.11	86.8 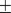 6.01	85.5 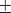 7.45	85.5 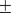 6.52
	**4** **s**	83.6 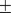 5.06	85.8 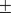 4.68	86.1 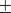 5.36	84.8 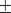 6.32	85.7 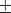 6.88	86.4 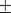 6.34
	**5** **s**	85.1 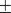 4.22	86.2 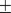 5.27	85.8 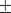 6.47	85.1 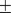 6.68	85.5 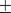 7.61	86.6 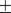 6.55

The percentage of impaired tissue number of voxels is 25%.

* NF: noise-free.

The classification rates that were determined using different percentages of abnormal voxels are presented in Table 3. The results demonstrated that a higher abnormal proportion produces a lower mean classification rate and a larger standard deviation. The mean classification rates decreased from a range between 78.2 and 90.1% with a 13% abnormal percentage to a range between 56.3 and 88.9% with a 50% abnormal percentage. Moreover, the standard deviations increased from at most 6.11% with a 13% abnormal percentage to 9.65% with a 50% abnormal percentage.

**Table 3:** The averaged classification rates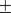 standard deviations (in percentage) for Monte Carlo simulations resulted from the EM-MoMG method initialized by the results of HC on the whitened simulated data with different delayed time, dispersion and various abnormal percentage under the condition of SNR = 70.

		Delay					
Percentage	Dispersion	0 s	1 s	2 s	3 s	4 s	5 s
**50%**	**0** **s**	–	56.3 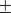 2.55	60.1 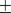 6.97	73.7 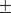 8.07	81.3 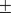 7.52	85.3 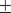 8.14
	**1** **s**	58.1 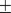 5.55	66.6 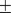 8.71	77.0 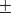 6.96	82.8 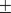 7.51	84.9 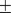 8.41	87.9 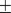 8.85
	**2** **s**	63.4 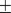 8.95	74.6 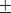 7.04	81.3 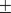 7.76	84.5 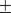 8.71	86.2 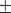 9.65	88.7 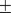 8.68
	**3** **s**	70.5 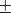 8.48	77.7 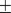 8.31	83.4 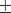 8.07	86.4 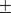 8.14	86.9 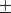 9.43	87.2 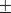 9.48
	**4** **s**	75.9 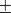 8.05	81.4 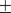 8.52	84.6 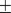 8.30	86.5 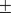 8.58	87.9 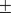 9.05	88.9 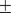 9.00
	**5** **s**	78.3 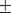 8.06	81.9 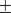 7.67	86.0 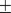 8.83	86.8 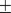 9.00	87.3 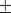 9.26	88.2 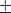 9.08
**25%**	**0** **s**	–	69.2 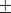 4.84	77.7 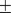 5.63	85.0 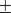 5.14	87.3 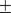 5.62	88.2 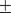 6.25
	**1** **s**	75.0 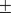 5.14	82.8 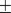 4.24	86.0 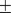 5.16	88.6 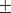 5.11	88.8 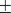 5.92	86.3 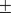 7.33
	**2** **s**	82.1 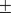 4.82	85.3 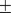 5.05	87.4 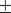 4.57	88.9 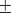 5.49	87.1 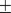 6.01	86.7 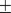 6.99
	**3** **s**	85.2 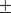 4.52	86.3 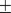 4.65	87.4 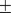 4.59	87.7 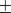 6.50	86.8 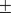 7.38	87.3 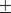 6.83
	**4** **s**	85.6 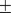 4.94	86.4 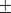 4.39	87.8 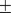 5.38	86.5 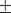 6.26	86.6 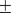 7.47	86.5 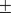 7.53
	**5** **s**	86.6 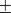 4.62	87.3 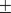 4.68	87.6 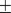 5.77	87.8 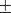 6.50	86.6 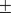 7.18	86.7 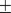 6.90
**13%**	**0** **s**	–	78.2 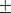 4.89	83.8 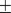 4.59	89.2 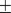 3.35	89.5 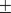 4.35	90.0 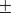 3.77
	**1** **s**	82.0 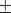 4.05	88.0 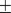 2.24	89.7 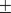 3.77	89.7 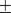 3.73	90.1 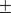 3.62	89.0 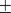 4.39
	**2** **s**	87.4 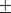 2.74	89.1 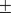 3.49	89.2 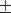 4.19	89.5 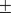 3.68	89.1 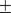 4.93	88.8 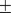 5.32
	**3** **s**	89.0 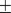 2.92	89.1 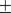 3.94	89.3 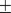 3.89	89.0 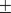 3.86	88.5 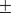 5.25	88.4 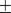 5.01
	**4** **s**	89.1 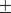 3.41	88.9 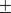 4.58	89.9 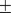 3.04	88.9 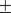 4.58	89.0 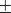 4.52	88.5 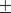 5.04
	**5** **s**	89.3 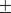 3.49	89.1 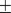 3.63	89.0 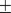 3.88	88.8 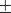 4.02	88.3 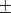 5.10	88.1 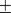 6.11

The abnormal percentage was the ratio of number of abnormal voxels to total number of voxels in a tissue type.

In the case of abnormal percentage = 50%: numbers of voxels in dArtery, dGM and dWM are 551*50%
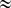
275, 1741*50%
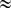
870 and 1636*50% = 818, respectively. Other two cases can be calculated in the same manner by replacing percentage of 50% with 25% and 13%, respectively.

### Classification Results from Normal Participants

Data from the five normal participants were processed and their perfusion images segmented into different compartments based on minimizing MDL values. Various tissue types, such as artery, GM, WM, vein+sinus, sinus, CSF+cp, CSF, vein with noise, sinus with noise, as well as artifacts, were segmented from normal data sets. The ratios of GM to WM for rCBV, rCBF, and MTT were 2.196±0.097, 2.259±0.119, and 0.968±0.023, respectively. These ratios were in agreement with other published reports [3], [27], [28].

### Results from a Patient with Right Internal Carotid Artery (ICA) Stenosis

**Figure 4:**
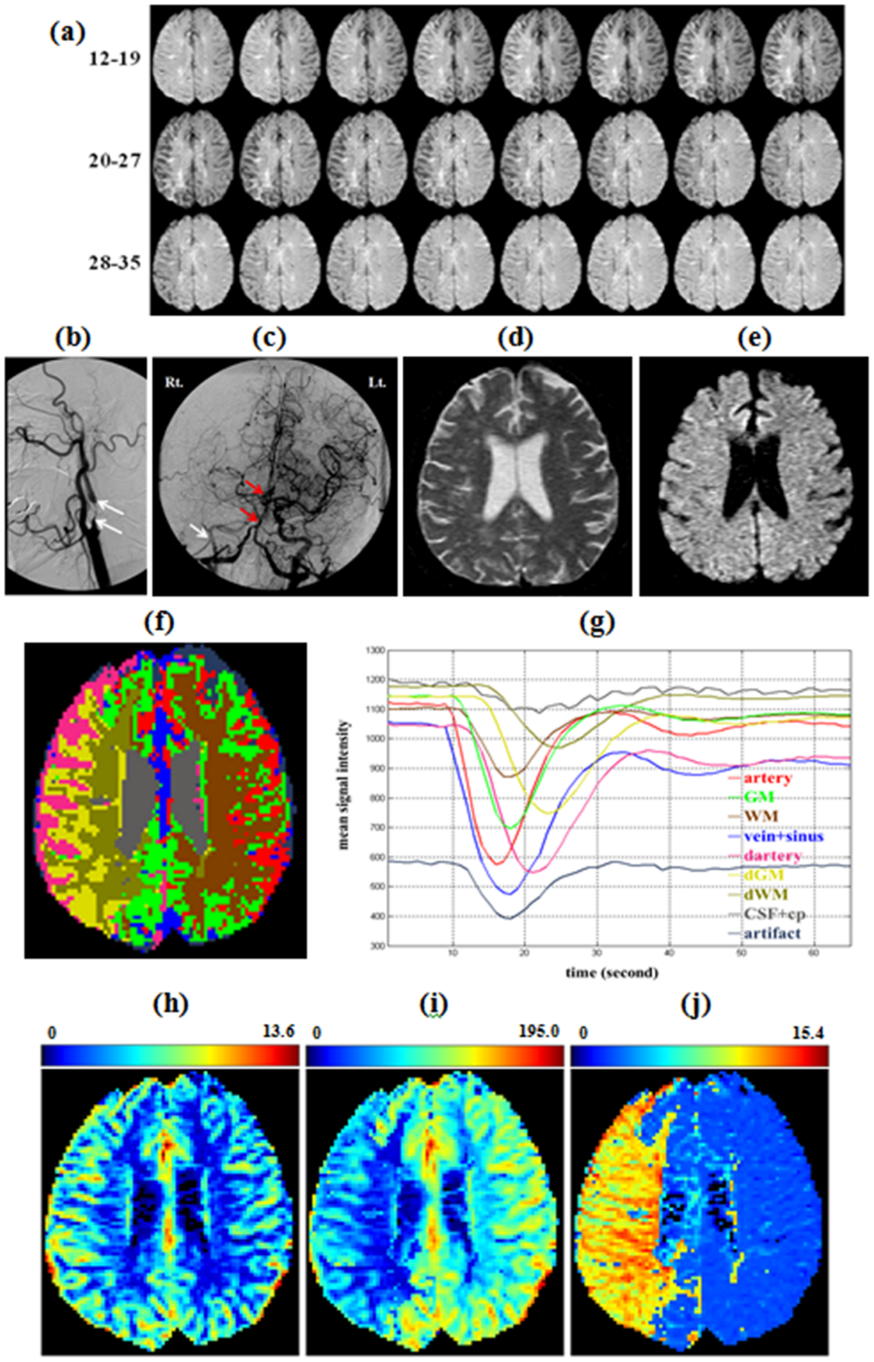
Segmentation results using the HC-EM-MoMG method for a patient with ICA stenosis. (a) Consecutive trans-axial DSC-MRI (12^th^ to 35^th^ second). (b) Right-lateral neck angiogram illustrated 99% stenosis on the right ICA labeled by the white arrows. (c) Another cerebral angiogram in anterior-posterior projection at the early arterial phase with the contrast injection from the aortic arch. The white arrow pointed out the delayed perfusion on the right ICA. (d) A trans-axial T_2_-weighted MR image at the similar slice location with the DSC-MRI. (e) A trans-axial Diffusion-weighted image confirms no infarct or stroke on the slice. (f) Segmented results shows nine hemodynamic components, namely artery (red), gray matter (green), white matter (brown), CSF and cp (gray), right ICA stenosis induced dArtery (pink), dGM (yellow), dWM (light brown), vein and sinus (blue), and artifact (dark blue). (g) The averaged signal-time curves of corresponding tissues. (h) rCBV map (scale unit is ml⋅100 g*^−^* ^1^). (i) rCBF map (scale unit is ml⋅100 g*^−^* ^1^⋅min*^−^* ^1^). (j) MTT map (scale unit is second).

The stenosis subject was a 78 year-old male subject with a right-side ICA 99% stenosis. A right-lateral neck angiogram of the subject showed the high-degree stenosis on the right ICA (white arrows in Fig. 4(b)). Another cerebral angiogram with an anterior-posterior projection at the early arterial phase, and with contrast injection from the aortic arch, was provided to illustrate the delayed perfusion on the right ICA. The right hemisphere of the brain exhibited delayed circulation (white arrow in Fig. 4(c)). This delayed perfusion was observed on consecutive trans-axial DSC-MR images (see Fig. 4(a)). The subject’s right hemisphere (abnormal side; left side of the images) had a signal drop and recovery between the 14^th^ and 28^th^ images, whereas the left hemisphere (normal side; right side of images) had a signal drop and recovery between the 12^th^ and 22^nd^ images, i.e., the signal drop occurred 2 s earlier and recovery occurred 6 s earlier on the normal side than on the stenosis-affected side. In addition, a trans-axial T_2_-weighted MR image (Fig. 4(d)) and a diffusion-weighted image (Fig. 4(e)) at a similar slice location as the DSC-MR image confirmed that there was no evidence of infarct or stroke on the slice. Segmented results (Fig. 4(f)) from the DSC-MR image using HC-EM-MoMG shows nine compartments, namely artery (red), GM (green), WM (brown), CSF+cp (gray), right ICA stenosis induced delayed/dispersed artery (pink), delayed/dispersed GM (yellow), delayed/dispersed WM (light brown), vein+sinus (blue), and artifact (dark blue). In Fig. 4 (f), it is worth noting that the GM and WM component maps cross the midline from the normal side to the stenosis side, especially in the anterior and posterior regions. Those results support the clinical findings that the subject’s anterior and posterior communicating arteries in the circle of Willis were intact. The averaged signal-time curves and corresponding spatial maps (Fig. 4(g) and (f)) demonstrate that tissues with similar characteristic perfusion profiles can be grouped by the EM algorithm. The results suggest that the impaired parts (dArtery, dGM and dWM) can be discerned from their contralateral counterparts (artery, GM and WM) by applying the HC-EM-MoMG method. One should note that the delayed or dispersed arterial input function (dAIF) obtained from the signal-time curve of dArtery was used to calculate the rCBF for dGM and dWM for minimizing the error induced by the delay and dispersion whereas the AIF obtained from the normal artery was used for other normal tissues such as GM, WM, CSF+cp, and vein+sinus. Fig. 4(h)–(j) exhibit hemodynamic-parametric maps (rCBV, rCBF and MTT) and the details for each of the classified tissue types are given in Table 4. The results show evidence of delay and dispersion in the impaired compartments. In particular, the TTP of the dGM (23.31
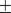
2.44 s) was larger than that of the normal GM (17.85
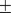
1.23 s), implying that there was delayed perfusion on the stenosis side. Moreover, the rCBF of the dGM (39.51
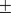
15.32 ml⋅100 g*^−^* ^1^⋅min*^−^* ^1^) was lower than that of the normal GM (54.26
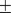
21.12 ml⋅100 g*^−^* ^1^⋅min*^−^* ^1^), and the MTT of the dGM (9.16
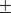
1.86 s) was significantly longer than that of the normal GM (5.85
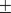
0.94 s), suggesting the presence of a dispersive effect from the ICA stenosis.

**Table 4:** The hemodynamic parameters of segmented tissue types for the patient with unilateral ICA stenosis.

	artery	GM	WM	CSF+cp	dArtery	dGM	dWM	vein+sinus
**TTP (second)**	16.44 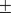 1.03	17.85 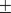 1.23	17.91 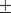 1.11	22.48 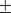 8.33	21.44 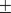 2.02	23.31 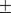 2.44	24.33 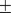 2.56	17.68 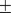 3.78
**rCBV (ml⋅100 g** *^−^* ^**1**^ **)**	7.05 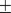 3.76	5.34 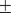 2.36	2.55 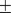 1.02	1.41 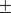 1.11	9.09 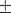 4.27	6.06 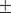 2.64	2.96 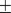 1.08	11.40 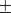 6.12
**rCBF (ml⋅100 g** *^−^* ^**1**^ **⋅min** *^−^* ^**1**^ **)**	74.74 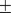 42.08	54.26 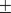 21.12	27.45 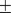 9.91	11.06 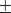 8.18	57.82 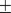 24.15	39.51 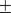 15.32	19.54 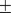 6.60	96.59 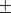 55.89
**MTT (second)**	5.64 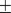 1.02	5.85 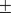 0.94	5.52 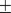 0.82	6.02 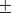 2.45	9.30 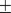 1.88	9.16 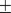 1.86	9.08 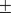 1.56	7.28 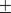 1.73

### Results from a Patient with Moyamoya Disease

**Figure 5:**
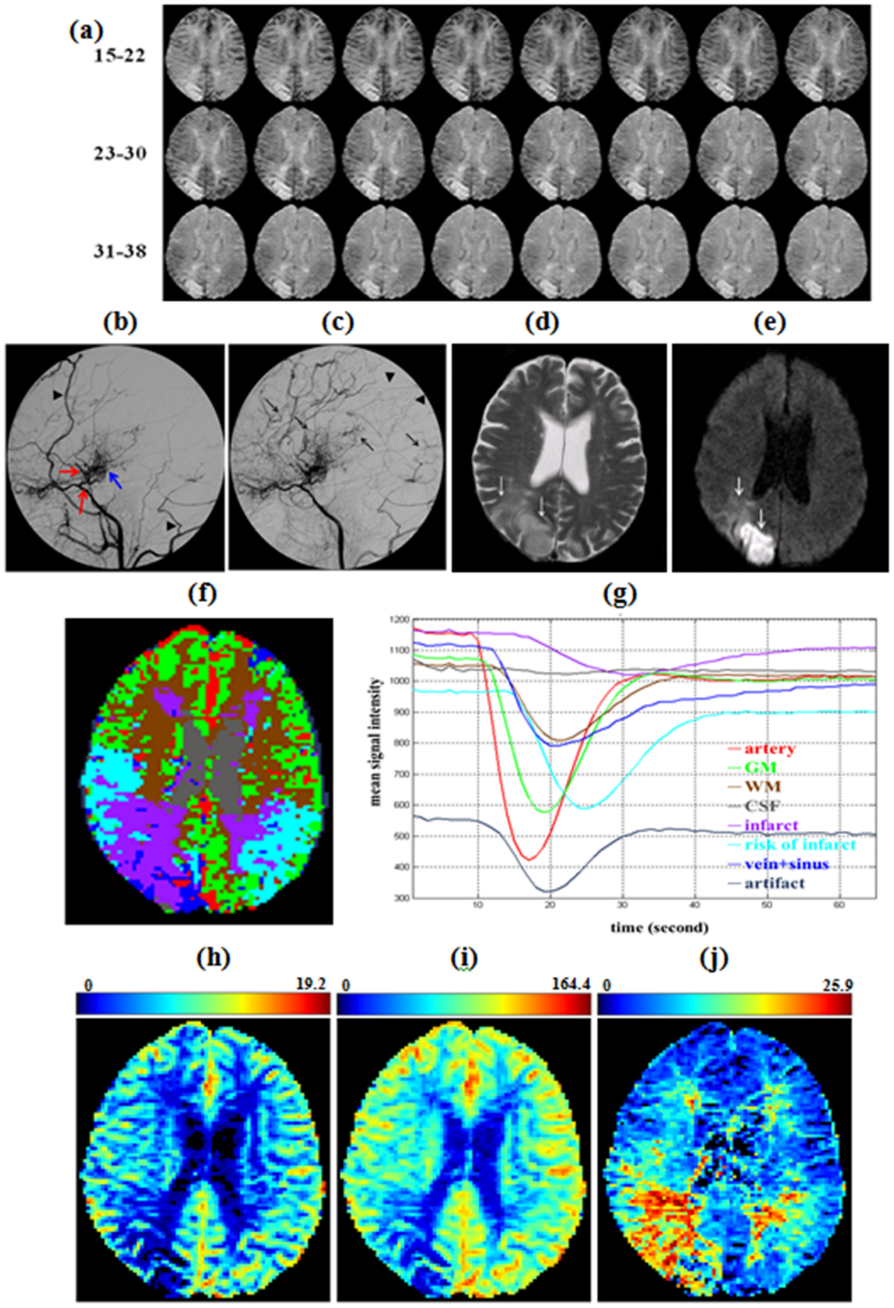
Segmentation results using the HC-EM-MoMG method for a patient with moyamoya disease. (a) Consecutive trans-axial DSC-MRI (15^th^ to 38^th^ second). (b) Right cerebral angiography at early arterial phase on lateral projection shows stenosis of the distal internal carotid artery, proximal segments of the anterior and middle cerebral arteries (red arrows). The moyamoya vessels are seen in the basal ganglia region (blue yellow). The prominent branches of external carotid artery (arrowheads) form the extracranial collaterals. (c) Another arterial phase angiogram taken at 1.5 seconds after (a) shows the late arrival blood supply (arrows). Her right parietal region is less blood-irrigated (arrowheads). (d) A trans-axial T2-weighted MR image shows abnormal high signals in her parietal-occipital region (arrows). (e) Diffusion-weighted image confirms an acute infarct with diffusion restriction (arrows). (f) Segmented results shows eight hemodynamic components, namely artery (red), gray matter (green), white matter (brown), CSF (gray), ischemic or infarct area (purple), areas supplied by collateral arteries with risk of infarct (cyan), vein and sinus (blue), and artifacts (dark blue). (g) The averaged signal-time curves of corresponding tissues demonstrated that the artery arrived earliest with maximum T2* signal drop followed by the GM or vein+sinus, WM or CSF, risk of infarct, and infarct. (h) rCBV map (scale unit is ml**⋅**100 g*^−^* ^1^). (i) rCBF map (scale unit is ml**⋅**100 g*^−^* ^1^⋅min*^−^* ^1^). (j) MTT map (scale unit is second).

Another case was a 57 year-old female patient with moyamoya disease. She suffered from sudden vision loss on her left side for 3 days. Her right cerebral angiogram at the early arterial phase with lateral projection showed stenosis of the distal internal carotid artery, proximal segments of the anterior and middle cerebral arteries (red arrows in Fig. 5(b)). Intracranial collateral arteries, so-called moyamoya vessels, are seen in the basal ganglia region (blue arrow in Fig. 5(b)). The prominent branches of external carotid artery (arrowheads in Fig. 5(b)) form the extracranial collaterals. Another arterial phase angiography taken at 1.5 seconds later than Fig. 5(b) shows the late arrival blood supply (arrows in Fig. 5(c)) via the collaterals. Her right parietal region is less blood-irrigated (arrowheads in Fig. 5(c)). The full perfusion could be shown by the consecutive trans-axial DSC-MRIs (see Fig. 5(a)). In addition, a trans-axial T2-weighted MR image shows abnormal high signals in her parietal-occipital region (arrows in Fig. 5(d)) and a diffusion-weighted image confirms an acute infarct with diffusion restriction (arrows in Fig. 5(e)). Segmented results (Fig. 5(f)) from DSC-MRI shows eight hemodynamic components, namely artery (red), gray matter (green), white matter (brown), CSF (gray), ischemic or infarct area (purple), areas supplied by collateral arteries with risk of infarct (cyan), vein and sinus (blue), and artifacts (dark blue). The averaged signal-time curves (Figs. 5(g)) demonstrated that the artery arrived earliest with maximum signal drop followed by the GM or vein+sinus, WM or CSF, risk of infarct, and infarct. Infarct area, mainly the right parietal-occipital brain, was the last to receive blood flow and shows much smaller signal drops compared to other areas. The resulting right occipital infarct as seen in Figs. 5(d) and (e) may be explained by the hemodynamic aberration shown in the infarct area (purple). Whereas the risk of infarct area presented the averaged signal-time curve with delay and dispersion effects compared to that of the GM tissue, and the spatial map of the risk of infarct resided near to the infarct area. In addition, using the classified regions (Fig. 5(f)) as masks on parametric images (Figs. 5(h)-(j)), the hemodynamic parameters, (rCBV, rCBF, MTT), for compartments artery, GM, WM, CSF, infarct, risk of infarct, and vein+sinus, can be easily calculated, respectively (see Table 5). The infarct area presented much lower rCBV (1.46
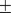
0.93 ml**⋅**100 g*^−^* ^1^) and rCBF (9.95
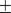
5.37 ml**⋅**100 g*^−^* ^1^⋅min*^−^* ^1^) but higher MTT (8.50
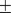
2.63 s) in comparison with that of GM and WM areas. In contrast, the risk of infarct area performs relatively toward normal rCBV (4.49
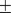
1.83 ml**⋅**100 g*^−^* ^1^) and rCBF (29.98±12.68 ml**⋅**100 g*^−^* ^1^⋅min*^−^* ^1^), which implies this area is worth in therapy to recover. These values can be re-computed after treatment to assess therapeutic effects.

**Table 5:** The hemodynamic parameters of segmented areas for the patient with moyamoya disease.

	Artery	GM	WM	CSF	infarct	risk of infarct	vein+sinus
**TTP (second)**	17.40 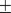 1.29	19.03 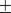 1.61	21.20 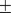 2.01	21.94 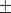 9.18	29.71 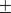 6.18	24.63 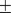 2.22	22.96 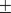 5.73
**rCBV (ml⋅100 g** *^−^* ^**1**^ **)**	7.05 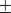 2.86	4.71 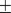 2.13	2.67 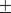 1.00	0.35 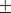 0.41	1.46 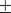 0.93	4.49 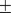 1.83	5.61 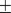 3.64
**rCBF (ml⋅100 g** *^−^* ^**1**^ **⋅min** *^−^* ^**1**^ **)**	63.93 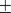 28.64	43.59 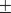 18.61	20.18 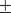 7.58	3.28 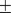 2.41	9.95 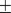 5.37	29.98 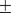 12.68	35.36 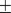 29.05
**MTT (second)**	6.70 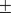 1.00	6.45 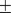 1.05	7.90 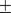 1.37	6.78 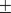 3.30	8.50 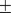 2.63	9.04 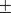 1.78	10.46 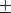 4.12

## Discussion

In this study, we improved the EM method by using the results of HC on whitened data to initialize the model parameters used in the MoMG model. It is worth noting that performing HC on whitened data is equivalent to conducting clustering on the independent component images resulting from FastICA [10]. To clarify, let the zero-mean-normalized and whitened data be 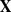. In a matrix of such data, each row represents an image and each column encodes the temporal information for each voxel, which can be regarded as a feature vector in the clustering process. In addition, let the optimal rotation matrix in the FastICA method be denoted by 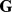. The resultant independent component images that manifest one major tissue type in each image are therefore denoted by 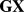. Since any rotation matrix holds the property 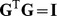, where the superscript ‘T’ stands for the matrix transpose, it follows that the Euclidean distance between any pair of feature vectors, namely, 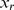 and 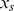, remains the same after rotational transformation, i.e., 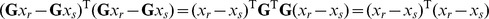. This shows that clustering on 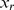
’s is the same a clustering on 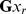
’s.

Monte Carlo simulations were carried out to compare the performance using HC results from whitened data and the random sampling of columns from the matrix representing the PCA data. The results of HC on the whitened data produced superior data clustering in terms of accuracy and variance than that obtained from random sampling on PCA data. That superiority was due to the similarity of scales in the corresponding components in any pair of column (feature) vectors from the whitened data. Through that similarity, each component in the subtraction of each paired vectors contributed equally to the calculation of Euclidean distance. On the contrary, in PCA data, the first components were the dominant terms in any pair of column vectors, and they overwhelm the contributions from the remaining components, producing results in which the features from the rest of components of each column vector have been suppressed. Thus, clusters resolved from the dissimilarity matrix can be better discriminated based on the whitened data. In brief, the simulation results suggest that the HC-EM-MoMG method performed well, particularly when the SNR was higher than 40.

Various tissue types, such as artery, GM, WM, vein+sinus, sinus, CSF+cp, CSF, vein with noise, sinus with noise, as well as artifacts, were segmented from normal data sets. Additionally, impaired tissue types, namely, dArtery, dGM, dWM, were segmented from an ICA subject’s data, and the infarct and risk of infarct were distinguished from others normal tissues in the moyamoya data. Since the slice locations from each of the normal subjects were not exactly the same, the segmented tissue types and the optimal number of clusters (between 7 and 8) varied slightly among subjects. Nevertheless, the artery, GM, WM, CSF+cp (or CSF) could be consistently segmented from the five normal data sets and the delivery of the contrast agent appeared in the following order: artery, GM, WM, and CSF+cp (or CSF).

The proposed HC-EM-MoMG method successfully showed spatial and temporal hemodynamic patterns for each of the dissected tissue compartments. Three advantages of using the proposed method are as follows. First, the averaged signal-time curve for arterial tissue can be used as an AIF; in particular, the dAIF can be identified in the subject with an ICA stenosis. That allows calculation of the hemodynamic parameters of abnormal tissues, which can be used to compensate for delay and dispersion effects. In the ICA stenosis subject, for example, the rCBF values of the dGM and dWM obtained from the normal AIF were 46.21
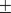
17.75 and 22.90
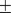
8.06, respectively, whereas from the dAIF, the values were 39.51
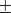
15.32 and 19.54
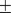
6.60, respectively. Second, in this method, voxels with similarly abnormal perfusion can be grouped into the same cluster, the corresponding signal-time curves and the hemodynamic parameters, namely rCBV, rCBF, MTT, and TTP, can be quantified for each tissue type, which can aid in diagnosis and therapeutic assessment. As presented in Fig. 4(f), the spatial location of each impaired compartment (dArtery, dGM, dWM) can be identified, and theirs hemodynamic parameters showed longer TTP and MTT, but lower rCBF compared to the normal tissues (see Table 4). In the case of moyamoya, the infarct area exhibited smaller signal drop and higher mean signal intensity of averaged signal-time curve which was similar to that of CSF, implying insufficient blood supply and death of brain tissue (Fig. 5(g)). This also can be observed with low rCBV and rCBF in infarct area. However, the hemodynamic parameters of risk of infarct, which were better than that of infarct area, presented lower rCBV, rCBF but longer TTP and MTT compared to GM. Although the risk of infarct exhibited the “toward-abnormal” states, it was potentially salvageable by clinical intervention for preventing enlargement of the infraction. The value of the recognition of risk of infarct area using HC-EM-MoMG method may facilitate the prognosis for treatment, which is similar to using MR perfusion-diffusion mismatch in identifying patients with acute ischaemic stroke for thrombolysis [29], [30]. Third, the segmented spatial maps derived from the method are useful when evaluating the hemodynamic compensation mechanism from collateral circulation through the circles of Willis; in particular, the integrity of the anterior communicating artery (AcoA), both side posterior communicating arteries (PcoA), and the basilar artery (BA) can be assessed. This compensation mechanism is illustrated in Fig. 4 (f) where the spatial distribution of the normal GM crosses the midline of two hemispheres within the anterior carotid artery (ACA) and posterior carotid artery (PCA) territories. As a result, the hemodynamics of these two areas on the stenotic side and in the normal GMs were grouped into one cluster, thereby suggesting the presence of perfusion compensation via the AcoA and BA. This result was supported by an angiogram assessment (red arrows in Fig.4 (c)) in which AcoA and BA patency was evident.

In future studies, the concept of the bargaining problem can be implemented for the optimisation of the parameters to assess the delay and dispersion effects. From the perspective of Game theory [31], the delay and dispersion parameters can be served as two players in the game, and the optimisation of these parameters can be achieved by the notion of an equilibrium point [32]–[34]. The optimisation of this two-player benefit in a non-zero-sum game is based on the optimisation of payoffs in the terms of game theory, where we expect to observe equilibrium in a set of alternatives in a spatial game theoretical framework [35]. Target recognition techniques, such as the cross-plot method [36], [37], can also be integrated for the automatic identification of tisse type after hemodynamic clustering using our proposed HC-EM-MoMG algorithm. Cross-plots of binary patterns (i.e., the binary maps of tissue templates) are exploered as image signatures for the observed target (i.e., the binary maps of tissue clusters) by capture of the spatial features with minimal computation [36], [37]. Some other segmentation mehods can be applied to facilitate the preprocessing of brain perfusion images, for example, an active contour model can automatically define the region of interest to remove the non-target image pixels, such as skull and scalp, or to locate the brain lesion, such as brain tumors [38].

In summary, we improved the EM method by using the results of HC on whitened data to initialize the parameters of MoMG models, termed as HC-EM-MoMG method. The results of Monte Carlo simulations confirmed that the mean classification rates of the proposed method can achieve over 93.6% and small standard deviations, which was superior to that using the random sampling as initializations. Moreover, this is the first study to assess the delay and dispersion effects on the hemodynamic segmentation, which is critical to the cerebrovascular diseases. Our results of Monte Carlo simulations showed that most classification rates of the HC-EM-MoMG method were greater than 80% whenever the differences in delay or dispersion between normal and corresponding impaired compartments were longer than one second. Finally, the analysis of data from the patients with unilateral ICA stenosis and moyamoya disease illustrated the effectiveness of the method for identifying impaired compartments. The proposed method can quantify impaired hemodynamics and serve as an aid in diagnosis and therapeutic assessment, such as evaluation of the integrity of the circle of Willis and identification of the risk of infarct area.
